# Analyzing stochastic transcription to elucidate the nucleoid's organization

**DOI:** 10.1186/1471-2164-9-125

**Published:** 2008-03-10

**Authors:** Alessandra Riva, Anne-Sophie Carpentier, Frédérique Barloy-Hubler, Angélique Chéron, Alain Hénaut

**Affiliations:** 1 Soluscience, Biopôle Clermont-Limagne, 63360 Saint-Beauzire, France; 2 Université Pierre & Marie Curie – Paris 6, UMR 7138 "SAE" CNRS UPMC MNHN ENS IRD, Case 05, 7 quai St Bernard, 75005 Paris, France; 3 CNRS-UMR 6026-Interactions Cellulaires et Moléculaires, Groupe B@sic, Université de Rennes 1, Campus de Beaulieu, 35042 Rennes, France

## Abstract

**Background:**

The processes of gene transcription, translation, as well as the reactions taking place between gene products, are subject to stochastic fluctuations. These stochastic events are being increasingly examined as it emerges that they can be crucial in the cell's survival.

In a previous study we had examined the transcription patterns of two bacterial species (*Escherichia coli *and *Bacillus subtilis*) to elucidate the nucleoid's organization. The basic idea is that genes that share transcription patterns, must share some sort of spatial relationship, even if they are not close to each other on the chromosome. We had found that picking *any *gene at random, its transcription will be correlated with genes at well-defined short – as well as long-range distances, leaving the explanation of the latter an open question.

In this paper we study the transcription correlations when the only transcription taking place is stochastic, in other words, no active or "deterministic" transcription takes place. To this purpose we use transcription data of *Sinorhizobium meliloti*.

**Results:**

Even when only stochastic transcription takes place, the co-expression of genes varies as a function of the distance between genes: we observe again the short-range as well as the regular, long-range correlation patterns.

**Conclusion:**

We explain these latter with a model based on the physical constraints acting on the DNA, forcing it into a conformation of groups of a few successive large and transcribed loops, which are *evenly *spaced along the chromosome and separated by small, non-transcribed loops.

We discuss the question about the link between shared transcription patterns and physiological relationship and come to the conclusion that when genes are distantly placed along the chromosome, the transcription correlation does not imply a physiological relationship.

## Background

### Terminology

There are many definitions for "gene expression". Some consider it a synonym of "transcription", others as the process from "gene to protein", including transcription, translation and, if applicable, any modifications of transcript and translational product.

For clarity's sake, we will talk in this article about "transcription data" and "transcription correlation", as the microarrays measure the relative abundance of mRNA transcripts. We will avoid, where possible, the term "(gene) expression".

We talk about large and small DNA "loops". Big loops are stretches of DNA with the diameter of the nucleoid which are available for transcription. The small loops have a smaller diameter and lie inside the nucleoid. How these are organized *in detail *physically (in terms of e.g. supercoiling), is a question we do not ask, as it is beyond the scope of the present work.

### Stochastic transcription and noise

As Samoilov et al. [1] point out, noise tends to be seen as something negative, which should be kept to a minimum and if possible eliminated. This is true for most of the fields where man is concerned. In biology, when taking readings of signals, it is indeed important to minimize the sources of noise coming from e.g. inaccurate reading settings.

However, sometimes noise deserves to be paid some attention. Gene transcription and translation and the biochemical reactions that take place between gene products are subject to stochastic fluctuations [2]. In transcriptomic analyses, signals below a certain threshold level tend to be classified as noise and are often discarded. It is presumed – correctly – that the signal does not originate from an "active" or "deterministic" transcription process and that it is therefore non-informative.

This conclusion, though, is wrong. The advent of single cell transcription analysis has shown that the random activation of genes, the random creation and destruction of messenger RNA can lead to the production of proteins that can be crucial in the cell's survival. An example is the stochastic activation of the competence gene in *B. subtilis*, part of the organism's stress response. In recent years researchers have started to examine this phenomenon and its repercussions on the cell more closely; we refer the interested reader to the works by Raser and O'Shea [2] and by Samoilov et al [1] for two comprehensive reviews on the subject of noise, stochasticity and phenotype.

### Studying transcription patterns to decode the nucleoid's organization

Despite varied and numerous approaches, little is known about the organization of the bacterial chromosome [3,4], partly because the system is a dynamic one, making direct observations difficult.

The advent of a new technology offers the opportunity to look at an old problem from a new and different point of view. It might confirm, confute or add new hypotheses.

Indeed, since their arrival at the end of the 1980s [5,6], microarrays have been used to explore the chromosomal organization at a small scale (DNA stretches tens to hundreds of bps long) or large scale (thousands of bps long) [7-9].

The basic idea is that genes that share transcription patterns, must share some sort of spatial relationship, even if they are not close to each other on the chromosome. One particular approach consists in gathering as many datasets from the literature as possible, pool them together and treat them as just one large data set, an approach that has given positive and encouraging results [8,10,11]

In a previous work we applied this technique to two phylogenetically widely different bacteria, *E. coli *and *B. subtilis *[12].

For both bacteria we analyzed the transcription patterns and found for all genes that "the co-expression of genes varies as a function of the distance between the genes along the chromosome" [12].

We found short-range correlations, thought to correspond to DNA turns on the nucleoid surface (14–16 genes), but also long-range correlations at well-defined distances. Surprisingly, these long-range correlations were found for all the genes, regardless of their localisation on the chromosome. In other words, picking any gene at random, its expression will be correlated with genes at well-defined distances.

This suggests an organization of the chromosome beyond that of operons.

Taking the solenoid model of the chromosome as the starting point, we suggested that the chromosome is organized into two different types of loops: large loops (with the nucleoid's diameter), corresponding to expressed stretches of DNA and accounting for the short-range correlations observed, and small loops (with a smaller diameter than the nucleoid), corresponding to non-expressed DNA.

NB: at the time we made no distinction between genes that are only transcribed and those that are also translated, using the term "expression" in its wider sense.

We had, however no explanation for the regular, long-range correlations observed.

The fact that the observations were made with such different organisms, suggested that they might show us a general property of double stranded, circular bacterial DNA.

### The aim of this paper

The aim of this paper is to examine the transcription correlations when the only transcription taking place is stochastic. In other words: when no active but only stochastic transcription occurs, can we observe any patterns in the transcription correlations? Do we find short- and, more interestingly, long-range correlations? And if so, how do these compare to the "active transcription" situation? Could the results be used to refine the model of the nucleoid organization? What can be said about the relationship between shared transcription patterns and physiological relationship?

To this end, we examined two particular sets of transcription data of *Sinorhizobium meliloti*.

#### The data sets

In set A all three replicons – the chromosome, pSymA and pSymB – are actively transcribed.

In set B, only the chromosome and pSymB are transcribed actively. pSymA only shows the stochastic transcriptional activity, a situation made possible by the fact that the plasmid does not contain any genes essential to the cell's viability under usual laboratory conditions (see below).

The analysis of the transcription data of pSymA in the two data sets should therefore allow us to answer the questions posed above.

#### A note on *S. meliloti*

*S. meliloti *is a nitrogen-fixing alpha-proteobacterium. It is distributed world-wide in many soil types, both in association with legumes or in a free-living form [13] and is used as a model species for the study of plant-bacteria symbiosis. Its genome contains 6206 ORFs distributed in three replicons: a chromosome of 3.65 Mb and two well-studied megaplasmids pSymA and pSymB, of 1.35 Mb and 1.68 Mb, respectively.

The smallest replicon, pSymA is specialized for nodulation and nitrogen fixation. It has been successfully cured without noticeable effects of bacterial viability in usual laboratory conditions [14], demonstrating that this replicon is not essential for cell viability (in the laboratory). Under certain culturing conditions, none of the proteins encoded for by the plasmid are transcribed, as revealed by enzyme assays.

pSymB contains several genes, which make it essential for cell viability, and several features suggest that it should be considered a chromosome rather than a plasmid [15].

## Results

The aim of this work is to see how the co-transcription intensities, or correlations, vary in function of the inter-gene distance along the megaplasmid pSymA when it is actively transcribed (data set A) and when only the stochastic transcription takes place (data set B).

We calculated the Kendall tau coefficient for all pairs of genes in the replicon and then measured the variation of this coefficient as a function of the gene distance using a linear autocorrelation function (see Methods).

We then submitted the autocorrelation function to a spectral analysis, in other words we decomposed the signal (the measured autocorrelations in function of gene distance) into the periods that make it up.

To determine whether the spectra obtained in the two data sets differed from each other from a statistical point of view, we compared them using the Mann-Whitney two-tailed test.

To obtain an internal control and enable us to eliminate any regularities observed that are created by chance, we performed the same calculations on the plasmid with a random permutation of its genes.

The same procedure was applied to the chromosome and the megaplasmid pSymB of *S. meliloti *(data not shown, see additional file 1).

### The autocorrelations

**Figure 1:**
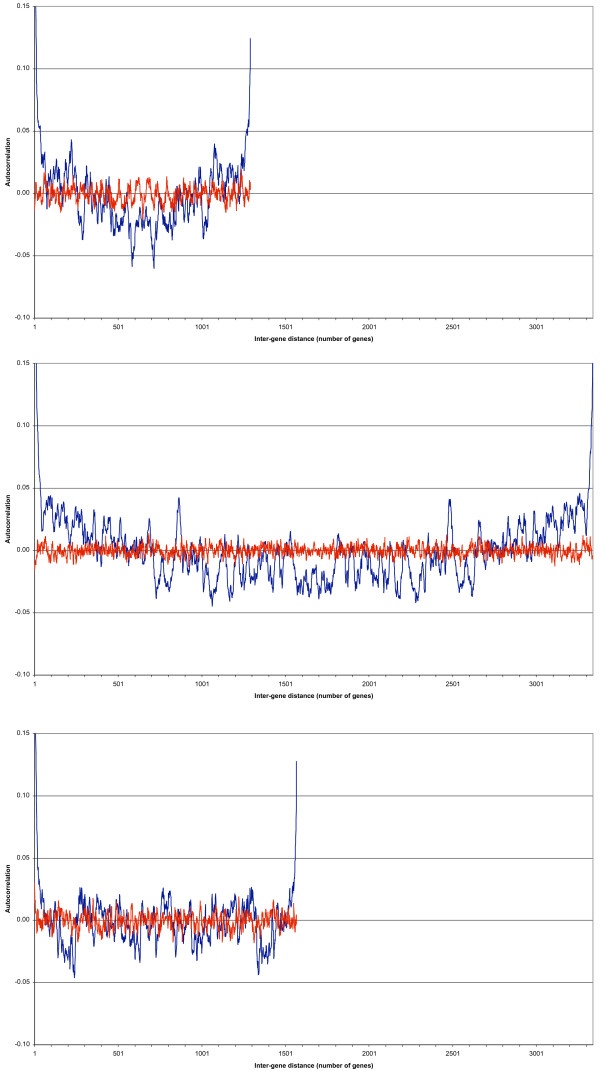
**a, b and c – The autocorrelation functions for data set A, when all three replicons are actively transcribed and translated**. Figure 1a shows the autocorrelation function for the megaplasmid pSymA (blue curve) when the plasmid is actively transcribed (and translated; data set A). The red curve shows the autocorrelation function when the genes' positions were randomly assigned. The Y-axis has been cropped at an autocorrelation of 0.15 for a clearer visual interpretation; the blue curve starts at an autocorrelation of 0.27 for a gene distance of one. Maxima (positive correlation) and minima (negative or anti-correlation) can be clearly distinguished, with a strong anti-correlation for genes that lie opposite of each other on the chromosome (a gene distance of around 650). Similarly, Figures 1b and 1c show the results for the chromosome and the megaplasmid pSymB, respectively, when both are actively transcribed (and translated; data set A). As can be seen, the autocorrelation functions for the three replicons are similar. Note: The figures are all at the same scale to better illustrate the different sizes of the three replicons. All Y-axes have been cropped at a value of 0.15 for (visual) clarity's sake.

Figure 1a shows the autocorrelation function for the megaplasmid pSymA (blue curve) when the plasmid is actively transcribed (and translated; data set A). The red curve shows the autocorrelations when the genes' positions were randomly assigned.

The blue curve starts at a value of 0.27 and then drops steeply, meaning that when a gene is transcribed, so will be its immediate neighbouring genes. A first local minimum is at a gene distance of about 45. Maxima (positive correlation) and minima (negative or anti-correlation) can be clearly distinguished, with a strong anti-correlation for genes that lie opposite of each other on the chromosome (a gene distance of around 650).

The curve has thus a similar and comparable behaviour to those of *E. coli *and *B. subtilis*.

As a comparison, Figures 1b and 1c show the results for the chromosome and the megaplasmid pSymB, respectively, when both are actively transcribed (and translated; data set A). As can be seen, the autocorrelation functions for the three replicons are similar, enforcing the idea that the observations made show us a general property of transcribed double stranded, circular bacterial DNA.

**Figure 2:**
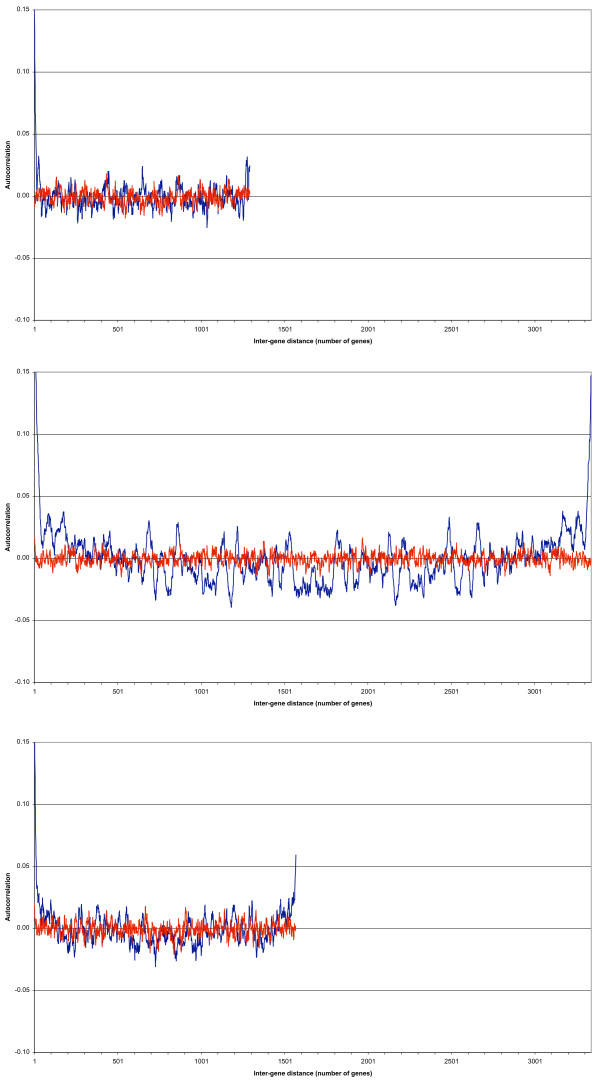
**a, b and c – The autocorrelation functions for data set B, when pSymA is transcribed stochastically only**. Figure 2a shows the autocorrelation function for the megaplasmid pSymA (blue curve) when only stochastic transcription takes place (data set B). The red curve shows the autocorrelation function when the genes' positions were randomly assigned. The Y-axis has been cropped at an autocorrelation of 0.15 for a clearer visual interpretation; the blue curve starts at an autocorrelation of 0.15 for a gene distance of one. The signal becomes quickly confounded with the noise (red curve). There are minima and maxima that stand out, but only a spectral analysis can tell, whether these are significant or not. Again to serve as comparison, Figures 2b and 2c show the autocorrelation functions for the chromosome and the megaplasmid psymB, respectively, for data set B. Both replicons are actively transcribed and translated, unlike psymA, and their autocorrelation functions are comparable to those in data set A (as confirmed by the spectral and statistical analyses, see additional file 1). Note: The figures are all at the same scale to better illustrate the different sizes of the three replicons. All Y-axes have been cropped at a value of 0.15 for (visual) clarity's sake.

Figure 2a shows the autocorrelations function for the megaplasmid pSymA (blue curve) when the only transcription is stochastic (data set B). The red curve shows the autocorrelations when the genes' positions were randomly assigned. The blue curve starts off at lower value (0.15) compared to data set A. This is not surprising, as we are looking at a situation where no active, but only stochastic transcription takes place. The signal is thus expected to be weaker.

As above, the data show that when a gene is transcribed, its immediate neighbours will be, too.

However, the first local minimum is already at a gene distance of 15 after which the signal becomes confounded with the noise (red curve). There are minima and maxima that stand out, but only a decomposition of the signal (the spectral analysis) and its statistical analysis can tell, whether these are significant or not.

Again to serve as comparison, Figures 2b and 2c show the autocorrelation functions for the chromosome and the megaplasmid pSymB, respectively, for data set B. Both replicons are actively transcribed and translated, unlike pSymA, and their autocorrelation functions are comparable to those in data set A (as confirmed by the spectral and statistical analyses, see additional file 1).

### The spectral analysis of pSymA

Table 1 shows the spectral analyses for the autocorrelations of pSymA (pSymA) and of pSymA with the genes' positions randomly permutated (rnd pSymA), for data set A and set B. Shown are the first twenty periods with the highest amplitudes, in descending order and the number of periods contained on the replicon.

**Table 1:** The spectral analyses of the autocorrelations

**Data set A (active transcription)**						**Data set B (stochastic transcription)**					
pSymA (set A)			rnd pSymA (set A)			pSymA (set B)			rnd pSymA (set B)		
			
No of Periods	Period	Amplitude	No of Periods	Period	Amplitude	No of Periods	Period	Amplitude	No of Periods	Period	Amplitude
1	1294.000	0.584	23	56.261	0.028	12	107.833	0.040	615	2.104	0.051
12	107.833	0.158	341	3.795	0.026	6	215.667	0.022	371	3.488	0.021
6	215.667	0.081	563	2.298	0.024	70	18.486	0.020	566	2.286	0.018
7	184.857	0.077	118	10.966	0.019	47	27.532	0.018	296	4.372	0.017
21	61.619	0.050	262	4.939	0.019	43	30.093	0.017	393	3.293	0.015
43	30.093	0.048	640	2.022	0.016	107	12.093	0.016	99	13.071	0.015
3	431.333	0.042	248	5.218	0.014	238	5.437	0.016	541	2.392	0.014
70	18.486	0.039	380	3.405	0.014	134	9.657	0.016	430	3.009	0.013
47	27.532	0.036	212	6.104	0.014	9	143.778	0.015	278	4.655	0.013
2	647.000	0.033	39	33.179	0.013	50	25.880	0.015	584	2.216	0.013
8	161.750	0.032	637	2.031	0.013	424	3.052	0.014	610	2.121	0.013
166	7.795	0.025	635	2.038	0.013	48	26.958	0.014	537	2.410	0.013
82	15.780	0.025	21	61.619	0.011	10	129.400	0.013	249	5.197	0.012
5	258.800	0.025	325	3.982	0.011	144	8.986	0.013	407	3.179	0.012
17	76.118	0.023	301	4.299	0.011	2	647.000	0.013	317	4.082	0.011
4	323.500	0.022	636	2.035	0.010	220	5.882	0.012	168	7.702	0.011
30	43.133	0.022	141	9.177	0.009	524	2.469	0.011	386	3.352	0.011
42	30.810	0.017	589	2.197	0.009	53	24.415	0.011	18	71.889	0.010
29	44.621	0.016	318	4.069	0.009	138	9.377	0.011	238	5.437	0.010
11	117.636	0.016	339	3.817	0.009	171	7.567	0.011	471	2.747	0.010

The table shows the spectral analyses for the averaged auto-correlations of pSymA (pSymA) and of pSymA with the genes' positions randomly permutated (rnd pSymA) for data set A and set B. Shown are the first twenty periods with the highest amplitudes, in descending order and the number of periods contained on the replicon. The two spectra of the "real" plasmid are made up of short and long periods. Both have, for example, periods of 108 and 206 genes. The random permutations, however, have only very short periods. The amplitudes for pSymA in set B are lower compared to the set A, as we are looking at a random phenomenon, which is necessarily weaker than active transcription. "No of periods": how many periods can be fitted along the replicon in question.

What is immediately noticeable is that the two spectra of the "real" plasmid are made up of short and long periods. Both have, for example, periods of 108 and 216 genes. The random permutations, however, have only very short periods.

Note, that for pSymA in data set A, the period of 1294 is responsible for the anti-correlation of genes lying opposite of each other along the chromosome, at a distance of about 650 genes (corresponding to half the period of 1294), seen in Figure 1a.

The amplitudes for pSymA in set B are lower compared to the set A. This should not surprise as we are looking at a random phenomenon, which is necessarily weaker than active transcription.

In order to verify that the spectra of pSymA truly are different from those obtained with the random permutations, we performed the Mann-Whitney two-tailed test. The results are shown in Table 2 and show that for both sets, the spectra of pSymA are clearly different from those obtained with the controls, where the gene order has been randomly permutated (p < 0.0001).

**Table 2:** Mann-Whitney two-tailed test for psymA

**Comparison**	**p-value**
(psymA set A) – (rnd psymA set A)	< 0.0001
(psymA set B) – (rnd psymA set B)	< 0.0001
(psymA set A) – (psymA set B)	0.006

In order to examine whether the various pairs of spectra (real replicon versus random permutation) differ from each other from a statistical point of view, we performed the Mann-Whitney two-tailed test. We also compared the spectra of the real replicon in the two datasets. For both, data set A and set B, the spectra of pSymA are clearly different from those obtained with the controls, where the gene order has been randomly permutated (p < 0.0001).

The comparison of pSymA in data set A versus data set B shows that though differing from a statistical point of view (p = 0.006), they are closer to each other than to the random permutations. This may be attributed to the presence of the long-range correlations in both sets.

The comparison of the spectra of pSymA in data set A compared to data set B shows that, though differing from a statistical point of view (p = 0.006), they are closer to each other than to the random permutations. This may be attributed to the presence of the long-range correlations in both sets.

We can thus say that the long-range correlations observed when active transcription takes place can also be observed when the transcription is stochastic only.

## Discussion

We demonstrate in this paper that even stochastic transcription shows the long-range correlation patterns previously observed in actively transcribed replicons: Given any gene, it will share its transcription patterns with other genes at well-defined distances.

The correlations observed are weaker compared to active transcription, but share the same distances.

The chromosome and megaplasmid pSymB of *S. meliloti *equally show (short- and) long-range correlations (see additional file 1 for the spectral and statistical analyses).

These distances vary between the bacteria (and within a bacterium between the different replicons): the distances observed in *E. coli*, *B. subtilis *and *S. meliloti's *three replicons are not identical, but of the same order of magnitude.

We can therefore confirm our hypothesis [12] that the observations made show us a general property of double-stranded circular bacterial DNA, chromosomal as well as plasmid.

By looking at a situation of purely stochastic transcription, we can eliminate all "outside" biological factors as the cause for the long-range observations made. We shall therefore look at the physical properties of the DNA.

### Can we explain the regular, well-defined long-range correlations?

We shall take the example of the megaplasmid pSymA when only stochastic transcription takes place, as it eliminates any role "outside" biological factors could play in active transcription.

We propose that the observations made can be explained with the physical properties of DNA. The DNA in a cell is in constant movement, it is mobile whilst the transcriptional and translational machineries are relatively immobile [16,17]. The DNA is subject to a number of physical constraints (a large molecule has to fit into a very finite space), as well as compaction and decompaction forces. These forces are in constant opposition. We refer the interested reader to the works by Woldringh and Nanninga [4] and Zimmerman [17].

Taking the solenoid model as a basis we had suggested that the chromosome is organized into two different types of loops: small ones, corresponding to non-transcribed DNA and large loops, corresponding to transcribed stretches of DNA lying on the nucleoid's surface and accounting for the short-range correlations observed [12].

Non-transcribed DNA is highly compacted, but being in constant movement, it can become locally unravelled and attached to the transcription machinery [17].

The data of this and our previous work suggest that the local decompaction will form a few, successive large DNA loops, which lie on the nucleoid's surface and have therefore its diameter. The number of successive large loops will be determined by the rigidity of the DNA, as well as the compaction and decompaction forces it is subjected to.

We now propose that these large loops are evenly spaced along the chromosome – in groups of a few at a time-, accounting for the long-range correlations: Big loops will cause the adjoining DNA to compact further, in a balancing of forces.

The most energy efficient way to accommodate large and small loops is to space the big loops (in packages of a few at a time) *evenly *along the replicon.

In other words, once a stretch of DNA is "trapped" in the transcriptional machinery, the DNA will compact and decompact itself in a way to adapt to this new conformation, and the most efficient way to do so, is by spacing the large loops of DNA evenly. These large loops of DNA thus created are transcribed, accounting for the long-range correlation patterns observed.

### Does transcriptional correlation imply physiological correlation?

We say that the reasons why genes have "colleagues" (genes with a shared transcription pattern) at certain well-defined distances are the physical constraints of the DNA.

Given this, are these "colleagues" linked by a same metabolic pathway, do they share some physiological aspect? In other words: has selection pressure "placed" related genes at these positions? Does transcriptional correlation automatically imply a physiological relationship?

The discussion about what drives mutations and gene order started with J.B.S Haldane in his paper "The Cost of Natural Selection" [18]. It continued for decades and we refer the interested reader to the work by Motoo Kimura and Tomoko Ota, "Theoretical Aspects of Population Genetics" [19], which gives a comprehensive introduction to the subject, the essence being, that most mutations are neutral (neither beneficial nor detrimental), and that it is highly unlikely that all the most advantageous genes can be reunited in a single organism.

The availability of an ever-increasing number of sequenced genomes has it made possible to study gene conservation and order amongst closely and distantly related organisms. The conclusions are that gene order is not conserved and rearrangements frequent, not only when comparing different bacterial species [20] but also different variants of the same bacterial strain [21]. Recently, Brinig et al. [22] analyzed 137 *Bordetella pertussis *strains (the agent of whooping cough) and found that although the gene content varied little, gene order varied significantly, suggesting a high amount of genome rearrangement in the species.

This flexibility in gene order is an additional, strong argument against the notion that genes, which are distant from each other along the chromosome but share transcription patterns, are necessarily related from a physiological point of view.

Genes are at the positions they are because of chance, not selection pressure. There are certainly exceptions, like the genes involved in the sulphur metabolism in *E. coli *[23] but for the great majority of genes that are not in close proximity of each other, a shared transcription pattern does not imply a shared physiological role.

This shared pattern is simply the result of the physical constraints of the DNA and chance: the creation of one big loop forces the DNA to re-adjust by the further compaction of certain stretches of DNA and the creation of other, regularly spaced big loops. The genes on the big loops are transcribed and they will therefore have a common transcription pattern. However, the functions of these genes can be wholly unrelated physiologically.

We should bare this in mind when searching for new metabolic pathways using transcription profiling.

## Conclusion

Transcription data can be used to elucidate the nucleoid's organization; in a previous work we had studied data from *E. coli *and *B. subtilis *and found that picking *any *gene at random, its transcription will be correlated with genes at well-defined short- and, more interestingly, long-range distances, without being able to account for these latter.

In this work, we analyze a particular set of transcription data of *S. meliloti*, which has allowed us to study the transcription correlations of exclusively stochastic transcription, in other words, when no transcriptional activators interfere with the DNA.

We observe again the short-range as well as the regular, long-range correlation patterns.

As no "outside" biological factors are involved in stochastic transcription, our explanation for the long-range correlations is based on the physical constraints acting on the DNA: once a stretch of DNA is "trapped" – by chance – in the transcriptional machinery, the DNA will compact and decompact itself in a way to adapt to this new conformation, by forming large (transcribed) and small (non-transcribed) loops. We had suggested that the DNA will form a few consecutive loops at a time, separated by small loops. We now propose that these groups of large loops are spaced *evenly*, at *regular distances *along the chromosome, this being the most efficient way, from an energetic point of view, to accommodate them. The large loops of DNA are all transcribed, at the same time, accounting for the long-range correlation patterns observed.

We argue that transcription correlation does not automatically imply a physiological relationship, as the genes are in the position they are mostly as a result of chance, rather than selection pressure. We should bare this in mind when searching for new metabolic pathways using transcription profiling.

## Methods

### Data used

We used transcription data of *S. meliloti*. Data set A is available at the EMBL-EBI ArrayExpress database (accession number E-TABM-73 and E-TABM-74). Data set B are unpublished microarray data of F. Barloy-Hubler's lab, available on request (please contact F. Barloy-Hubler).

### Procedure

The aim is to see how co-expression intensities (correlations) vary in function of the inter-gene distance. In other words we want to examine, whether the expression of any given gene is correlated to that of other genes, and if so, if the distances between these genes show any regularities, similar to the observations made in our previous work on *E. coli *and *B. subtilis*. The procedure has been described elsewhere [12], here a brief summary:

In order to obtain an internal control, we permutated the gene order for the three replicons at random and repeated the calculations described below. Any patterns observed with these random sets must be "subtracted" from those obtained with the real sets.

For each replicon (chromosome, pSymA and pSymB) and each data set (set A and set B) we evaluate the co-expression among each pair of genes with a non-parametric correlation: the Kendall tau.

To define the Kendall tau τ, we start with the *N *data points (*xi*, *yi*), the expression levels of the genes *x *and *y *in the experimental condition *i*, respectively. Considering all the *1/2N(N-1) *pairs of data points (*xi*, *yi*) (*xj*, *yj*), we call a pair "concordant" if the differences (*xi*-*xj*) and (*yi*-*yj*) have the same sign, and "discordant" if the differences have opposite signs. The Kendall's tau τ is a correlation of signs and the following simple combination of these various counts:

τ = (*concordant *- *discordant*)/(*concordant *+ *discordant*)

NB: Kendall also foresaw the highly unlikely event of *ex aequos *(*xi-xj *or *yi-yj *equal to zero) with a subsequent modification of the above formula, detailed in [12].

We calculate the autocorrelation function of the Kendall tau matrix (in other words the transcription correlation in function of gene distance) [24,25].

We submit the autocorrelation function to a spectral analysis, in other words we decompose the signal – the measured autocorrelations in function of gene distance-into the periods that make it up, retaining the first twenty periods with the highest amplitudes. We used XLSTAT-Pro/3DPlot/Time.

We compare the spectral analyses in pairs with the Mann Whitney two-tailed test, to determine any statistically significant differences between them.

## Authors' contributions

AR drafted the manuscript, ASC retrieved the data from the databases and performed the statistical analyses, FBH and AC obtained the remaining data, AH conceived the study, participated in its analysis and coordination. All authors participated in the elaboration of the model, read and approved the final manuscript.

## Supplementary Material

Additional file 1 **Riva additional data 1**. "Riva additional data 1" is a doc file. It contains all the results obtained in this study: the autocorrelation functions, spectral analyses and the Mann Whitney two-tailed test of the spectral analyses, for *S. meliloti's *three replicons. We have chosen to group the data into one file. The material contained in this file is not necessary to the understanding of the article, it only provides additional information. Click here for file
