# Supplementary material for: Analyzing stochastic transcription to elucidate the nucleoid's organization
Source: BMC Genomics. 2008 Mar 10;9:125. doi: 10.1186/1471-2164-9-125 (PMC2270832; doi:10.1186/1471-2164-9-125)
Supplement: Additional file 1 — Riva additional data 1. "Riva additional data 1" is a doc file. It contains all the results obtained in this study: the autocorrelation functions, spectral analyses and the Mann Whitney two-tailed test of the spectral analyses, for S. meliloti's three replicons. We have chosen to group the data into one file. The material contained in this file is not necessary to the understanding of the article, it only provides additional information. [file 1471-2164-9-125-S1.doc]

Additional Data

**-Spectral Analysis of the autocorrelation functions for the three replicons and both data sets.**

**Statistical Analysis of the Spectra**

In order to examine whether the various pairs of spectra (real replicon versus random permutation, set A versus set B) differ from each other from a statistical point of view, we performed the Mann-Whitney two-tailed test.

**Comparaison p-value**

(chr set A) - (chr set B) 0,427

(chr set A) - (rnd chr set A) < 0,0001

(chr set B) - (rnd chr set B) < 0,0001

(pSymA set A) - (pSymA set B) 0,006

(pSymA set A) - (rnd pSymA set A) < 0,0001

(pSymA set B) - (rnd pSymA set B) < 0,0001

(pSymB set A) - (pSymB set B) 0,036

(pSymB set A) - (rnd pSymB set A) < 0,0001

(pSymB set B) - (rnd pSymB set B) 0,001

(chr set A + B) - (pSymA set A) 0,966

(chr set A + B) - (pSymB set A) 0,576

(chr set A + B) - (pSymA set B) 0,001

(chr set A + B) - (pSymB set B) 0,047
